# Mutation of the LXCXE Binding Cleft of pRb Facilitates Transformation by ras *In Vitro* but Does Not Promote Tumorigenesis *In Vivo*

**DOI:** 10.1371/journal.pone.0072236

**Published:** 2013-08-06

**Authors:** Srikanth Talluri, Sarah M. Francis, Frederick A. Dick

**Affiliations:** 1 London Regional Cancer Program, Western University, London, Ontario, Canada; 2 Children’s Health Research Institute, Western University, London, Ontario, Canada; 3 Department of Biochemistry, Western University, London, Ontario, Canada; Texas A&M University, United States of America

## Abstract

**Background:**

The Retinoblastoma protein (pRB) is a key tumor suppressor that is functionally inactivated in most cancers. pRB regulates the cell division cycle and cell cycle exit through protein–protein interactions mediated by its multiple binding interfaces. The LXCXE binding cleft region of pRB mediates interactions with cellular proteins that have chromatin regulatory functions. Chromatin regulation mediated by pRB is required for a stress responsive cell cycle arrest, including oncogene induced senescence. The *in vivo* role of chromatin regulation by pRB during senescence, and its relevance to cancer is not clear.

**Methodology/Principal Findings:**

Using gene-targeted mice, uniquely defective for pRB mediated chromatin regulation, we investigated its role during transformation and tumor progression in response to activation of oncogenic ras. We report that the pRB^∆L^ mutation confers susceptibility to escape from HrasV12 induced senescence and allows transformation *in vitro*, although these cells possess high levels of DNA damage. Intriguingly, *LSL-Kras, Rb1* ^*∆L/∆L*^ mice show delayed lung tumor formation compared to controls. This is likely due to the increased apoptosis seen in the early hyperplastic lesions shortly following ras activation that inhibits tumor progression. Furthermore, DMBA treatment to induce sporadic ras mutations in other tissues also failed to reveal greater susceptibility to cancer in *Rb1* ^*∆L/∆L*^ mice.

**Conclusions/Significance:**

Our data suggests that chromatin regulation by pRB can function to limit proliferation, but its loss fails to contribute to cancer susceptibility in ras driven tumor models because of elevated levels of DNA damage and apoptosis.

## Introduction

Oncogene induced senescence (OIS) has emerged as a tumor suppressive mechanism that acts as a barrier to transformation *in vivo* [1]. Pre-malignant lesions such as melanocytic nevi and prostatic intraepithelial neoplasias (PIN lesions) in humans are found to be rich in cells expressing markers of senescence, while senescent cells are rarely found in the corresponding malignant stages [2–4]. In recent years, mouse models of human cancer have been generated that show activation of senescence in response to the expression of oncogenes [2]. Similar to human lesions, senescence in these mouse models is predominantly associated with pre-malignant stages of tumorigenesis suggesting a role for senescence in inhibiting or delaying tumor progression in response to oncogene activation *in vivo*.

Senescence is associated with activation of tumor suppressor pathways regulated by p53 and pRB proteins [5,6]. These two pathways coordinately inhibit the growth of pre-cancerous cells and prevent them from becoming tumors. Accordingly, disruption of these tumor suppressor pathways has been shown to result in increased susceptibility to tumorigenesis in response to oncogenes [4,7–10]. In mouse models, simultaneous activation of oncogenes such as HrasG12V or BRAFV600E combined with loss of p53 or p16, that act upstream of pRB, resulted in escape for OIS and malignant transformation [7–9]. Notably, enhanced tumor progression in these models strongly correlates with loss of oncogene induced senescence markers, further supporting the notion that escape from OIS is a prerequisite for malignant progression.

Senescence is a permanent cell cycle exit in which cells can remain in a state of arrest throughout the life span of the organism [5]. The permanence of a senescent arrest is partly attributed to the chromatin changes that repress transcription, and block DNA synthesis and cell division [11]. Senescent cells form heterochromatin bodies called senescence associated heterochromatic foci (SAHFs) that are proposed to encompass and silence proliferative genes [12]. SAHFs are enriched in repressive chromatin modifications such as H3K9me3 and H3K27me3 and several chromatin associated proteins that aid in chromatin compaction and transcriptional repression [12,13]. Defects in this heterochromatin assembly pathway have been shown to compromise the stability of a senescent arrest *in vitro* and are predicted to promote tumorigenesis *in vivo* [10]. Intriguingly, mice lacking Suv39h1, the enzyme capable of tri-methylating histone H3K9, show defective senescence in response to oncogenic stress and display increased susceptibility to tumorigenesis in the Eµ-Nras model that expresses oncogenic NrasG12D in the hematopoietic compartment [10]. However, whether or not the chromatin changes during senescence have a broader tumor suppressive role *in vivo*, in response to oncogene activation in different tissues is still an open question that needs further investigation.

Lung cancer is the leading cause of cancer related deaths in humans and the most common type of lung cancer is the pulmonary adenocarcinoma [14,15]. Activated K-Ras mutations are the most frequent genetic alteration and they are associated with approximately 30% of human lung adenocarcinomas [15,16]. Over the last two decades a number of mouse strains have been generated to model human lung adenocarcinomas and they have greatly aided our understanding of the progression of the disease, as well as the oncogenes and tumor suppressors that influence it [17]. Conditional mutant models were generated harboring a latent mutant allele of *KRas* (LSL-KrasG12D) at its endogenous locus that can be activated sporadically in the lung cells by administering a cre expressing adenovirus [18–20]. This model closely mimics the human disease and allows for investigation of the different stages of lung cancer progression.

The pRB tumor suppressor pathway is disabled in most human cancers [21]. Although mutations of *RB1* are rare in lung adenocarcinomas, *CDKN2A*, the gene encoding p16^INK4a^ the upstream activator of the pRB pathway is a frequently targeted for mutation [22]. p16^INK4a^ is a cyclin dependent kinase inhibitor (CKI) that inhibits D type cyclins associated with Cdk4 or Cdk6 in the G1 phase of the cell cycle, resulting in hypo-phosphorylation and activation of pRB in response to oncogenic insults. The pRB tumor suppressor protein acts by repressing E2F dependent transcription of genes involved in cell cycle progression both by direct binding and also through recruitment of chromatin regulatory complexes to these promoters [23]. pRB is one of the major effectors of senescence and both E2F inhibition and chromatin regulatory functions have been shown to be crucial for proper senescent arrest in cultured cells in response to activated oncogenes [11,12,24]. In the LSL-*KrasG12D* lung cancer model, complete lack of pRB is knoen to promote malignant transformation and enhance tumorigenesis [25]. However, the contribution of different aspects of pRB function to its tumor suppressive role is not clear as complete deletion of the *Rb1* gene has many consequences.

In order to investigate the role of chromatin regulation by pRB during oncogene induced senescence and tumorigenesis *in vivo*, we used a gene targeted mouse model in which the endogenous *Rb1* allele is mutated to encode a protein that is defective in binding to chromatin regulators [26]. This allowed us to study the role of chromatin regulation by pRB in isolation, as the pRB^∆L^ mutant is able to interact with and regulate E2F transcription factors similarly to wild type. In order to investigate what affect the *Rb1* ^*∆L*^ mutation might have on tumor susceptibility *in vivo*, in response to activated oncogenes, we crossed *Rb1* ^*∆L/∆L*^ mice with LSL-*KrasG12D* mice. We show that the *Rb1* ^*∆L*^ mutation allows escape for OIS and immortalization *in vitro*. However, these cells sustain extensive DNA damage. Surprisingly, the *Rb1* ^*∆L*^ mutation delays lung tumors in the LSL-*KrasG12D mice*. *LSL-Kras; Rb1* ^*∆L/∆L*^ compound mutant mice show fewer lung adenomas compared to LSL-*KrasG12D* mice alone following adenovirus-cre mediated activation of oncogenic Kras in the lung. Increased apoptosis in early stage, atypical hyperplastic lesions, correlates with reduced adenomas later during tumor development. We further show that chromatin regulation by pRB does not accelerate tumorigenesis in a chemical carcinogen model that is known to induce ras mutations. Taken together, our results suggest that loss of chromatin regulation by pRB facilitates escape from cell cycle arrest, but elevated levels of DNA damage and apoptosis prevent it from synergizing with oncogenic ras.

## Results

### The Rb1^*∆L*^ mutation promotes escape from OIS and transformation *in vitro*

**Figure 1:**
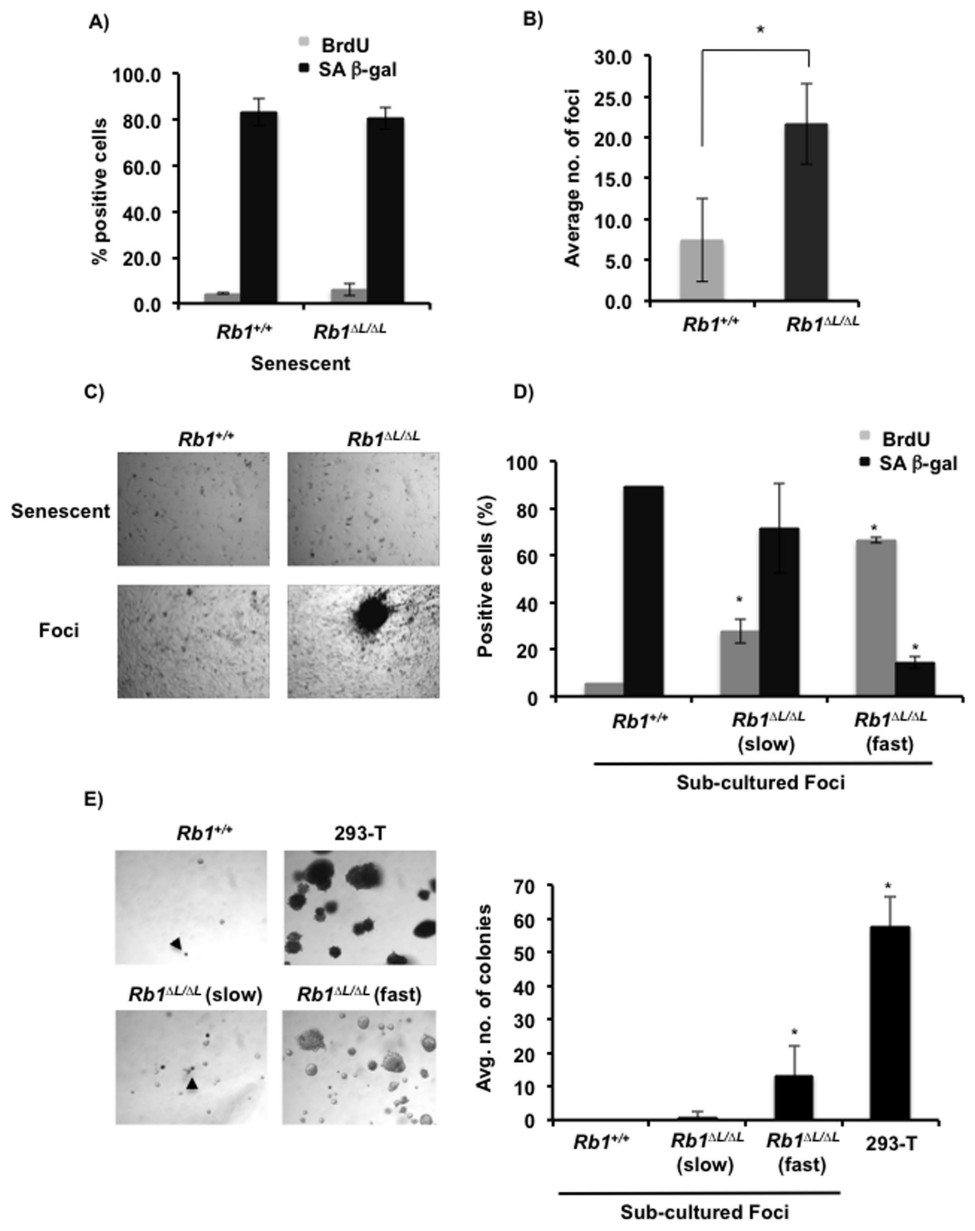
Escape from HrasV12 induced senescence and transformation in *Rb1* ^*∆L/∆L*^ MEFs. Asynchronously growing wild type and *Rb1* ^∆L/∆L^ MEFs are induced to senesce by retroviral mediated expression of oncogenic HrasV12. After 3 days of selection, cells were plated in medium and cultured for at least 10 days. All cells were pulse labeled with BrdU for 4 hours. (**A**) The percentage of BrdU and SA β-gal positive cells in senescent *Rb1* ^*+/+*^ and *Rb1* ^∆L/∆L^ cultures were determined. (**B**) Quantitation of foci from senescent *Rb1* ^*+/+*^ and *Rb1* ^∆L/∆L^ cultures. The average number of foci formed 3 weeks post selection were compared between genotypes. The mean number of foci was compared between genotypes using a t-test (* p<0.0004). (**C**) Phase contrast microscopy images of wild type and *Rb1* ^∆L/∆L^ cells either senescent (10 days post-selection) or foci (3 weeks post-selection). (**D**) The percentage of BrdU and SA β-gal positive cells in sub-cultured foci from senescent *Rb1* ^*+/+*^ and *Rb1* ^∆L/∆L^ cells was determined. *Rb1* ^∆L/∆L^ clones were grouped into slow growing (slow) or fast growing (fast) based on their proliferative capacity as determined by BrdU incorporation and SA β-gal staining. Mean values were compared with wild type controls using a t-test (* p<0.05). (**E**) Sub-cultured foci were also subjected to culture in soft agar to determine their capacity for anchorage independent growth. 293-T cells were used as a positive control. Quantitation after 2 weeks of culturing in soft agar is shown on the right. The average number of colonies/field was determined from 10 random fields and mean colony values were compared with wild type using a t-test. (* p<0.05).

Using mouse embryonic fibroblasts (MEFs) we previously reported that pRB-LXCXE binding cleft mediated interactions are required for heterochromatin assembly and stable repression of E2F target genes during senescence [24]. In response to oncogenic Hrasv12 expression, *Rb1* ^*∆L/∆L*^ MEFs undergo a defective senescence arrest that is characterized by elevated DNA synthesis and sensitivity to cell cycle re-entry in response to stimuli such as ectopic E2F1 expression. We wondered if the *Rb1* ^*∆L*^ mutation allows senescent *Rb1* ^*∆L/∆L*^ MEFs to escape permanently from oncogenic HrasV12 induced senescent arrest and immortalize. In order to test this, we induced senescence in asynchronously growing wild type and *Rb1* ^*∆L/∆L*^ MEFs by expressing oncogenic HrasV12 by retroviral transduction. HrasV12 expressing cells were selected for 3 days in puromycin containing medium following which they were re-plated at low density and cultured further in selection medium until they become senescent. 10 days after re-plating, most cells in both genotypes were senescent as determined by senescence associated β-galactosidase (SA-β-gal) staining and BrdU incorporation (Figure 1A). We then continued to culture the cells in puromycin containing medium to monitor spontaneous escape from senescence arrest. We quantified the number of spontaneous escape events by counting the distinct foci that appeared in these cultures. We counted a significantly higher number of foci in *Rb1* ^*∆L/∆L*^ cultures compared to the wild type cultures 3 weeks following HrasV12 expression (Figure 1B). The cells in these foci had lost the characteristic features of senescence, such as flattened morphology, vacuolated cytoplasm, and enlarged nuclei (data not shown). Interestingly, the foci in *Rb1* ^*∆L/∆L*^ cultures were bigger and often formed multilayered aggregates suggestive of loss of contact inhibition, characteristic of immortalized cell clones (Figure 1C). This suggests that the *Rb1* ^*∆L*^ mutation increases susceptibility to escape from oncogene induced senescence (OIS) and this might lead to spontaneous immortalization in culture.

In order to determine if these foci are composed of immortalized cells that have permanently escaped from senescence, and to study their growth characteristics, we isolated cells from foci from both the *Rb1* ^*+/+*^ and *Rb1* ^*∆L/∆L*^ cultures expressing HRasV12. We sub-cultured the cells extracted from the foci in puromycin containing medium and analyzed them for proliferation and senescence using BrdU labeling and SA-β-gal staining respectively. Interestingly, most of the clones recovered from *Rb1* ^*+/+*^ cultures failed to survive the sub-culturing process and all of them eventually arrested with features of senescence (Figure 1D). In contrast, we were able to successfully sub-culture about half the clones generated from *Rb1* ^*∆L/∆L*^ cultures. However, these clones showed varied growth properties prompting us to categorize them into slow growing and fast growing groups (Figure 1D). The slow growing clones still showed significantly higher BrdU incorporation compared to the cells from *Rb1* ^*+/+*^ clones (Figure 1D). Strikingly, the fast growing clones had a very high proportion of BrdU positive cells and very few cells stained positive for SA-β-gal. These cells showed a highly refractive spindle shaped appearance and had an increased metabolic rate as suggested by rapid acidification of culture medium (data not shown). This suggests that the *Rb1* ^*∆L/∆L*^ MEFs that escape from oncogenic HrasV12 induced senescent arrest have increased proliferative capability and show characteristic properties of immortalized cell clones.

We next performed soft agar colony formation assays to determine if the escaped clones are capable of anchorage independent growth, indicative of transformation *in vitro* (Figure 1E). We used 293-T cells as a positive control for our assay. After two weeks of culturing, most cells from the *Rb1* ^*+/+*^ clone failed to grow in soft agar. In contrast, cells from the fast growing *Rb1* ^*∆L/∆L*^ clones formed multicellular aggregates suggesting that they are capable of anchorage independent growth. Interestingly, although some of the cells from the slow growing *Rb1* ^*∆L/∆L*^ clones managed to form small aggregates in soft agar, we noticed cell death in these aggregates that seem to limit further growth. This suggested that in contrast to the *Rb1* ^*+/+*^ clones, *Rb1* ^*∆L/∆L*^ clones that had escaped from senescence were capable of anchorage independent growth. However, cell death likely limits the *in vitro* transformation potential of some of these immortalized clones.

Taken together, the experiments above suggest that the *Rb1* ^*∆L*^ mutation promotes escape from oncogenic HrasV12 induced senescence and immortalization *in vitro*. Some of these clones attain anchorage independent growth potential in soft agar, suggestive of transformation.

### The p53-p21 pathway limits the growth potential of escaped Rb1^*∆L/∆L*^ cell clones expressing HrasG12V

We wanted to further investigate the molecular basis of differential growth properties and *in vitro* transformation abilities of the sub-cultured foci that had escaped senescence and determine which other pathways might be limiting the transforming ability of these clones. Oncogenic ras has been shown to induce hyper proliferation and replication stress resulting in increased DNA damage [27–29]. Elevated DNA damage response (DDR) signaling results in the phosphorylation and activation of the p53 tumor suppressor protein. Loss of pRB function has previously been shown to result in p53 activation mediated by E2F1 and p19^ARF^ leading to increased apoptosis in response to oncogenic ras [30]. Given that the *Rb1* ^*∆L*^ mutation leads to deregulated E2F target gene expression during senescence [24], we wondered if the p53 pathway could act as a checkpoint to limit transformation of *Rb1* ^*∆L/∆L*^ cells expressing Hras.

**Figure 2:**
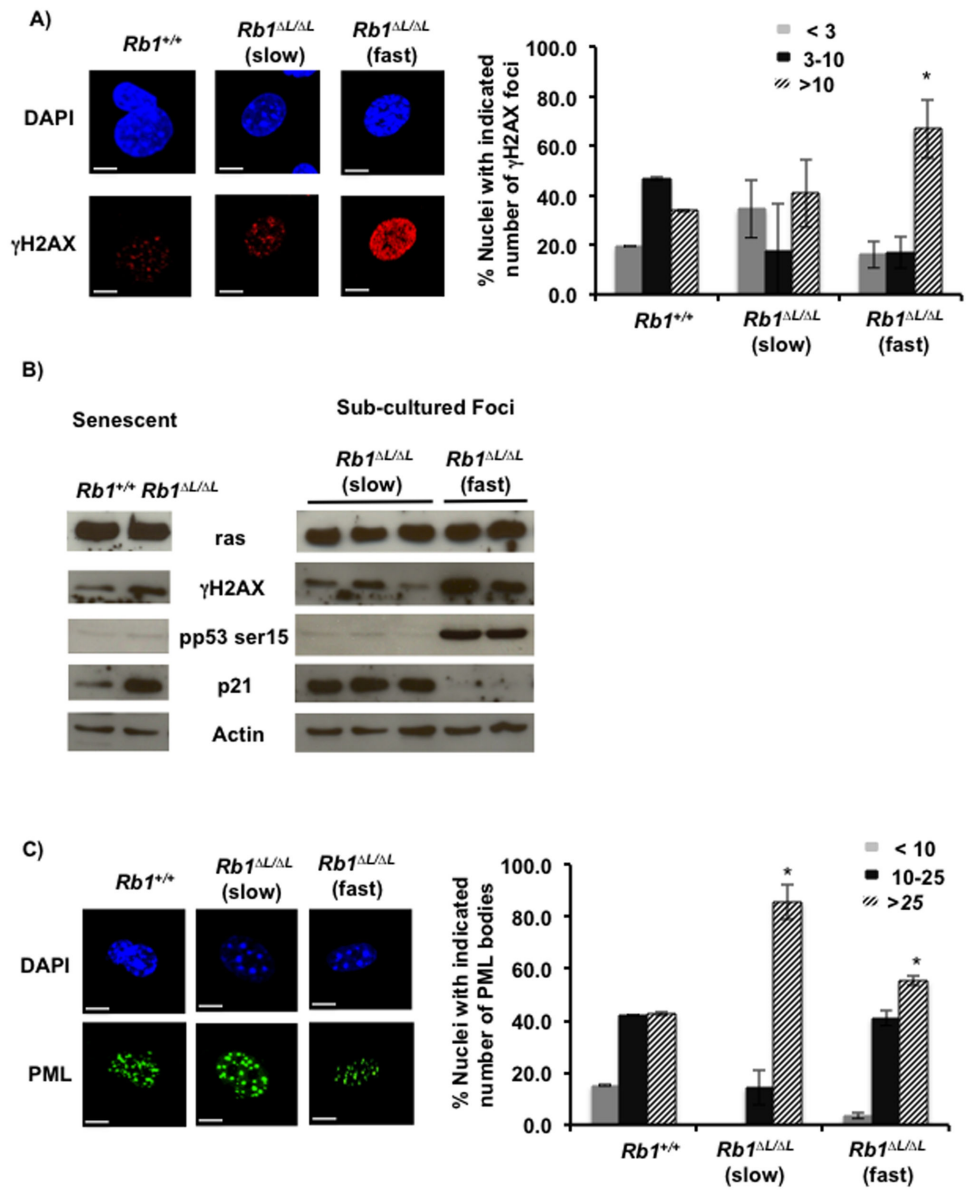
Increased DNA damage in *Rb1* ^*∆L/∆L*^ cells sub-cultured from foci. *Rb1* ^*+/+*^and *Rb1* ^∆L/∆L^ clones were sub-cultured from foci and stained with antibodies against γH2AX and PML. (**A**) Immuno-fluorescent staining of *Rb1* ^*+/+*^ and *Rb1* ^∆L/∆L^ clones with a γH2AX specific antibody to determine the extent of DNA damage. Quantitation is shown in the graph on the right. Asterisk indicates significant difference from wild type controls (t-test, p<0.05). Scale bars are 10µM. (**B**) Western blots for DNA damage response proteins in senescent *Rb1* ^*+/+*^ and *Rb1* ^∆L/∆L^ MEFs (left) and slow and fast growing *Rb1* ^∆L/∆L^ sub-cultured foci are shown. (**C**) Anti-PML antibody staining of *Rb1* ^*+/+*^ and *Rb1* ^∆L/∆L^ cells sub-cultured from foci. Quantitation of PML nuclear bodies is shown on the graph to the right. Asterisk indicates significant difference from wild type controls (t-test, p<0.05). Scale bars are 10µM.

We first analyzed whether DNA damage signaling is intact in *Rb1* ^*+/+*^ and *Rb1* ^*∆L/∆L*^ sub-cloned foci (Figure 2A and B). Escaped clones from both the *Rb1* ^*+/+*^ and *Rb1* ^*∆L/∆L*^ senescent cultures showed high levels of DNA damage as shown by the increased quantity of γH2AX foci/nucleus (Figure 2A). Strikingly, the fast growing clones have significantly higher levels of foci/cell (>10) compared to both the slow growing *Rb1* ^*∆L/∆L*^ and the *Rb1* ^*+/+*^ clones. Moreover, cells in the *Rb1* ^*∆L/∆L*^ clones, where γH2AX stained the entire nucleus, were suggestive of drastic DNA damage. We also analyzed γH2AX abundance by western blotting. As shown in Figure 2B we were able to detect γH2AX in senescent cells as well as all five sub-cultured clones that we tested. Interestingly, senescent *Rb1* ^*∆L/∆L*^ MEFs showed higher γH2AX levels and p21 induction compared to wild type cells, suggesting that the *Rb1* ^*∆L*^ mutation confers susceptibility to DNA damage and this results in increased activation of the p53-p21 pathway during the initial senescent arrest. Similar to immunofluorescent microscopy in Figure 2A, the fast growing *Rb1* ^*∆L/∆L*^ clones showed relatively higher levels of γH2AX compared to the slower growing clones by western blotting (Figure 2B). Furthermore, all clones showed similar expression of ras by western blotting. We wondered if the increased DNA damage results in the activation of the p53-p21 pathway in these clones. As shown in Figure 2B, we could detect phosphorylation of p53 at ser15 in slow growing sub-cultured clones and we interpret p53 to be active in these cells because we also detect p21 expression. Interestingly, in the fast growing *Rb1* ^*∆L/∆L*^ clones p53 is incapable of inducing the expression of p21 despite high levels of phosphorylation of p53 at ser-15. This indicates that p53 is functionally inactive in fast growing cells that have escaped a senescent arrest.

Oncogenic ras expression induces the expression of PML and assembly of PML nuclear bodies that are important for growth suppression in a p53 dependent manner [31,32]. PML has been shown to have an essential role during apoptosis and cooperates with p53 to induce apoptosis in response to DNA damage [30,33]. Consequently, we investigated the PML nuclear body formation in the escaped clones. All of the clones tested displayed abundant PML bodies in the nucleus that were similar in quantity to senescent cells. This suggests that the pathway upstream of PML body formation is still intact and the escape from senescence is due to defects downstream of PML body formation (Figure 2C).

These experiments show that the initial signaling events in response to the expression of oncogenic ras are still active in the cell clones that escape from senescence. The escaped clones accumulate DNA damage resulting in the activation of the p53-p21 pathway. *In vitro* transformation and anchorage independent growth in soft agar correlates with disruption of the p53-p21 pathway. Taken together, this indicates that defective senescence in *Rb1* ^*∆L/∆L*^ cells allows resumption of proliferation, but this is opposed by p53.

### The Rb1^*∆L*^ mutation does not enhance oncogenic ras driven cancer

**Figure 3:**
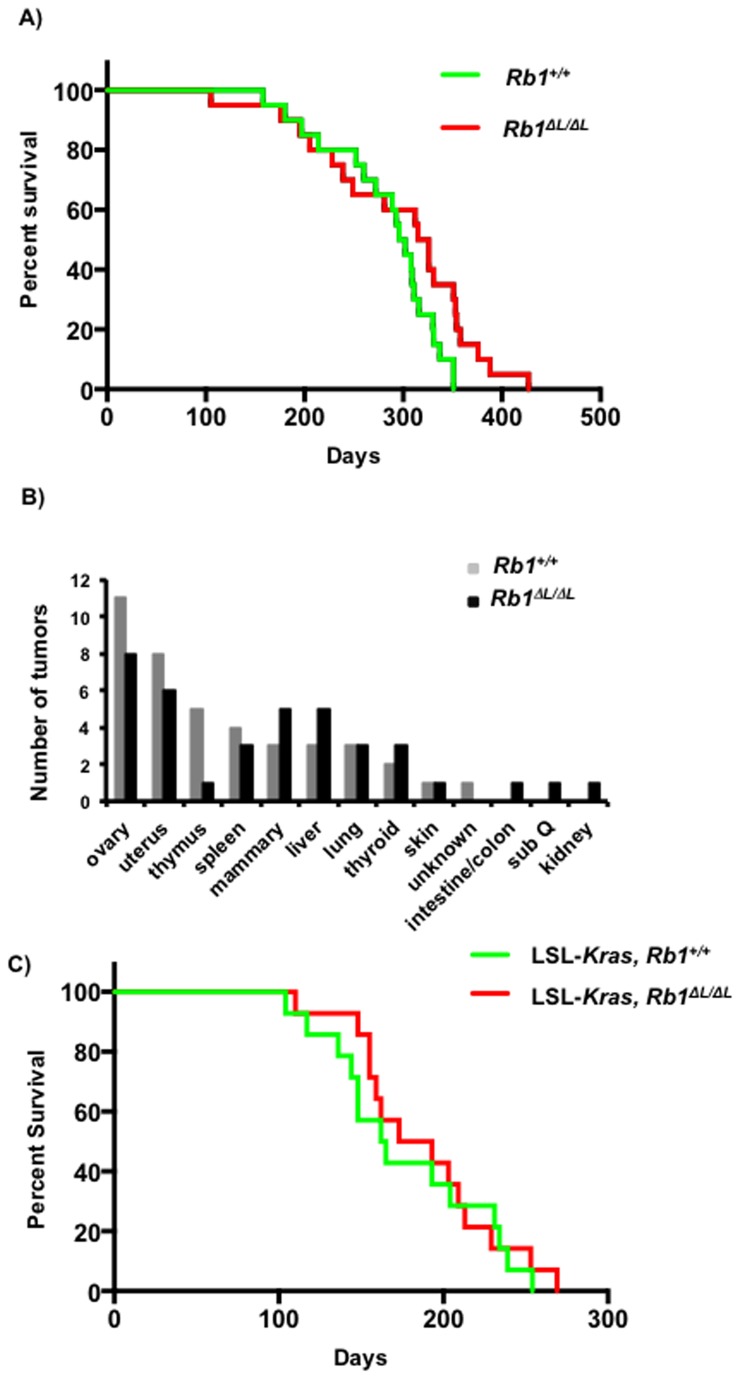
The *Rb1* ^*∆L*^ mutation does not affect tumor free survival in multiple models of ras induced cancer. Six to eight week old *Rb1* ^*+/+*^ and *Rb1* ^∆L/∆L^ mice were dosed with 1mg of 7,12-dimethyl benz[a]anthracine (DMBA) in canola oil by orogastric lavage weekly for 4 weeks. (**A**) Kaplan-Meier survival curves of *Rb1* ^*+/+*^ (n=20) and *Rb1* ^∆L/∆L^ (n=20) mice treated with DMBA. The median survival age is 299 for wild type and 320.5 days for *Rb1* ^*∆L/∆L*^ (p=0.0537, log rank test). (**B**) Quantification of tumor disease sites at the time of necropsy in *Rb1* ^*+/+*^ and *Rb1* ^∆L/∆L^ mice treated with DMBA. (**C**) Six to eight week old LSL-*Kras, Rb1* ^*+/+*^ and LSL-*Kras, Rb1* ^∆L/∆L^ mice were infected intra-nasally with Ad-Cre to activate the oncogenic K-rasG12D allele. The mice were monitored over time for tumor free survival. Kaplan-Meier survival curves LSL-*Kras, Rb1* ^*+/+*^ (n=14) and LSL-*Kras, Rb1* ^∆L/∆L^ (n=14) mice. The median survival age is 163.5 for control and 183 days for *Rb1* ^*∆L/∆L*^ (p=0.636, log rank test).

We used 7,12-dimethyl benz[a]anthracine (DMBA) induced chemical carcinogenesis to induce tumors. Administration of DMBA has been shown to cause ras mutations and promote tumorigenesis in several tissues in mouse models of cancer [34,35]. We wanted to investigate if the *Rb1* ^*∆L*^ mutation promotes tumorigenesis in response to DMBA treatment. We treated six week old *Rb1* ^*+/+*^ and *Rb1* ^*∆L/∆L*^ mice with DMBA once a week for four weeks by oral gavage and monitored the mice for tumors. As seen in Figure 3A, the *Rb1* ^*∆L*^ mutation did not significantly alter the tumor free survival of the mice in a DMBA induced tumor model. The overall tumor free survival rate was similar between the genotypes with 95% of *Rb1* ^*+/+*^ mice and 100% *Rb1* ^*∆L/∆L*^ mice succumbing to tumors. The median tumor free survival is 320.5 days for the *Rb1* ^*∆L/∆L*^ mice compared to 299 days for the *Rb1* ^*+/+*^ mice (p=0.0537, log rank test), indicating that *Rb1* ^*∆L/∆L*^ mice may actually be slightly resistant even though this difference doesn’t reach a 95% confidence interval. Necropsies of the mice from both groups revealed tumor burden in a broad range of tissues (Figure 3B). In addition, we did not observe a significant difference between the two groups in terms of disease site as both genotypes developed a broad range of cancer types. Given the preponderance of ras mutations that are known to be caused by DMBA, it suggests that the *Rb1* ^*∆L*^ mutation does not promote tumorigenesis in response to oncogenic ras.

To follow up on this surprising result, we switched to a well characterized oncogenic Kras induced lung cancer model, Lox-STOP-Lox-KrasG12D (henceforth referred to as LSL-*Kras*) [18,20]. In this system we can control the initiation of cancer through adenovirus cre (Ad-Cre) administration, and investigate progression to determine the effects of the *Rb1* ^*∆L*^ mutation on oncogenic ras driven cancer. We crossed LSL-*Kras* mice with *Rb1* ^*∆L/∆L*^ mice that are defective for LXCXE binding cleft mediated interactions to generate compound mutant mice (LSL*-Kras*, *Rb1* ^*∆L/∆L*^). We then induced the expression of the latent *Kras* allele in both the control mice (LSL*-Kras*, *Rb1* ^*+/+*^) and our compound mutant mice using Ad-Cre by intranasal infection. We monitored the mice following Ad-Cre infection for lung tumor free survival over a 40 week period, as well as progression at 6, 8, 10, 12, and 16 week time points.

Deletion of pRB has been previously shown to co-operate with oncogenic KrasG12D to promote tumorigenesis in this background [25]. These compound mutant mice develop more aggressive tumors and succumb to their tumors earlier than KrasG12D mice alone. We hypothesized that the *Rb1* ^*∆L*^ mutation would permit escape from senescence in lung tumor lesions, thus accelerating tumorigenesis. However, as shown in Figure 3C the *Rb1* ^*∆L*^ mutation did not significantly alter the tumor free survival of the oncogenic KrasG12D expressing mice. The median survival was 163.5 days following activation of oncogenic KrasG12D by Ad-Cre for the LSL*-Kras*, *Rb1* ^*+/+*^ mice compared to 183 days for the compound mutant LSL*-Kras, Rb1* ^*∆L/∆L*^ mice (Figure 3C). This suggested that, in contrast to our *in vitro* results where *Rb1* ^*∆L*^ promotes escape from oncogene induced senescence and transformation, the *Rb1* ^*∆L*^ mutation does not promote tumorigenesis or affect the tumor free survival of mice expressing oncogenic ras *in vivo*.

### Fewer lung tumor lesions in Rb1^*∆L/∆L*^ mice expressing oncogenic Kras

Oncogene induced senescence is widely believed to act as a barrier to transformation and cancerous growth *in vivo*, and expression of oncogenic ras has been shown to activate senescence thereby limiting tumorigenesis in mouse models [2]. Our *in vitro* results suggested that cells from *Rb1* ^*∆L/∆L*^ mice have defective senescence allowing them to escape from this arrest and transform. However, this did not lead to enhanced tumor susceptibility *in vivo*, as a result we wanted to further investigate tumorigenesis in the LSL*-Kras, Rb1* ^*∆L/∆L*^ mice more closely by quantifying the number of lesions that develop in response to *Kras* activation.

**Figure 4:**
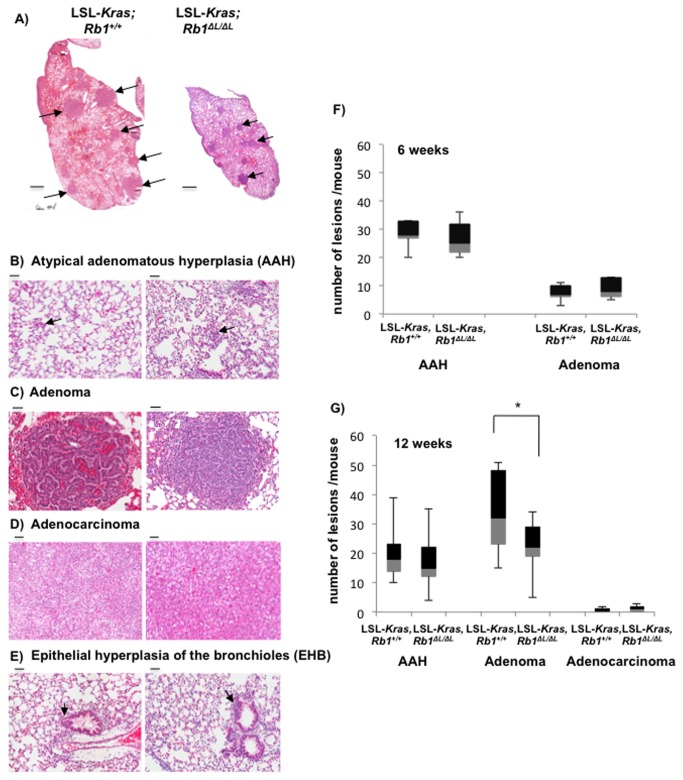
Fewer lung tumor lesions in *Rb1* ^∆L/∆L^ mice expressing KrasG12D. Six to eight week old LSL-*Kras, Rb1* ^*+/+*^ and LSL-*Kras, Rb1* ^∆L/∆L^ mice were infected intra-nasally with Ad-Cre to activate the oncogenic KrasG12D allele. The mice were euthanized at 6 and 12 weeks post Ad-Cre infection. (**A**) Haematoxylin and Eosin stained lung sections from 12 week old mice of the indicated genotypes are shown. Arrow heads indicate some of the lesions. Scale bars are 5 mm. (**B**) Microscopy images of H&E stained lung tissues to demonstrate atypical adenomatous hyperplasia (AAH) in both LSL-*Kras, Rb1* ^*+/+*^ and LSL-*Kras, Rb1* ^∆L/∆L^ mice. Scale bar is 50 µm. (**C**) H&E section of lung tissue to demonstrate adenomas in both genotypes of mice. Scale bar is 50 µm. (**D**) H&E section of lung tissue showing adenocarcinoma tissue. Scale bar is 50 µm. (**E**) Lung tissue section showing H&E staining of epithelial hyperplasia of the bronchioles (EHB). Scale bar is 50 µm. (**F**) Quantitation of the lung tumor lesion types in LSL-*Kras; Rb1* ^*+/+*^ and LSL-*Kras; Rb1* ^∆L/∆L^ mice at six weeks post Ad-Cre infection. Box and whisker plots show 25^th^ percentile, median and 75^th^ percentiles. Whiskers show highest and lowest values in the group. Means were compared by t-test. (**G**) The same analysis as in A, except at 12 weeks post Ad-Cre infection. Means were compared by t-test, an asterisk indicates a statistically significant difference (p<0.05).

We investigated lung tumor lesions from the LSL*-Kras*, *Rb1* ^*+/+*^ and LSL*-Kras, Rb1* ^*∆L/∆L*^ mice post activation of oncogenic *Kras* by Ad-Cre. At 12 weeks post adenovirus infection, we were able to detect the different types of lesions that define progression in this model and many were visible at a gross level (Figure 4A). Similar to previous reports in the literature [18], we detected atypical adenomatous hyperplasia (AAH), adenoma, adenocarcinoma, and epithelial hyperplasia of the bronchioles (EHB) in both the experimental groups (Figure 4B–E). AAH and adenoma are benign lesions and are considered precursors for adenocarcinoma. As shown in Figure 4F, lungs from LSL*-Kras, Rb1* ^*∆L/∆L*^ mice and LSL*-Kras*, *Rb1* ^*+/+*^ controls have similar levels of pre-malignant lesions at six weeks post Ad-Cre treatment. Strikingly, by 12 weeks there were significantly fewer adenomas in LSL*-Kras, Rb1* ^*∆L/∆L*^ mutants (Figure 4G). One possibility for the reduced number of adenomas seen in LSL*-Kras, Rb1* ^*∆L/∆L*^ mice at 12 weeks is accelerated progression to adenocarcinoma. Since we did not measure a concomitant increase in the number of adenocarcinomas in LSL*-Kras, Rb1* ^*∆L/∆L*^ mice compared to controls we think this explanation is unlikely. We also did not detect any difference between the two groups in the number of AAH lesions, the precursor of adenoma, at either time point. Interestingly, at the time of harvesting the lungs for histology, we routinely observed that the lungs from LSL*-Kras, Rb1* ^*∆L/∆L*^ mice were smaller in size compared to the control LSL*-Kras*, *Rb1* ^*+/+*^ mice at 12 weeks (Figure 4A). Taken together, this suggested that, tumor initiation levels were similar, but that the *Rb1* ^∆L^ mutation ultimately impedes the growth of *Kras* induced tumors.

**Figure 5:**
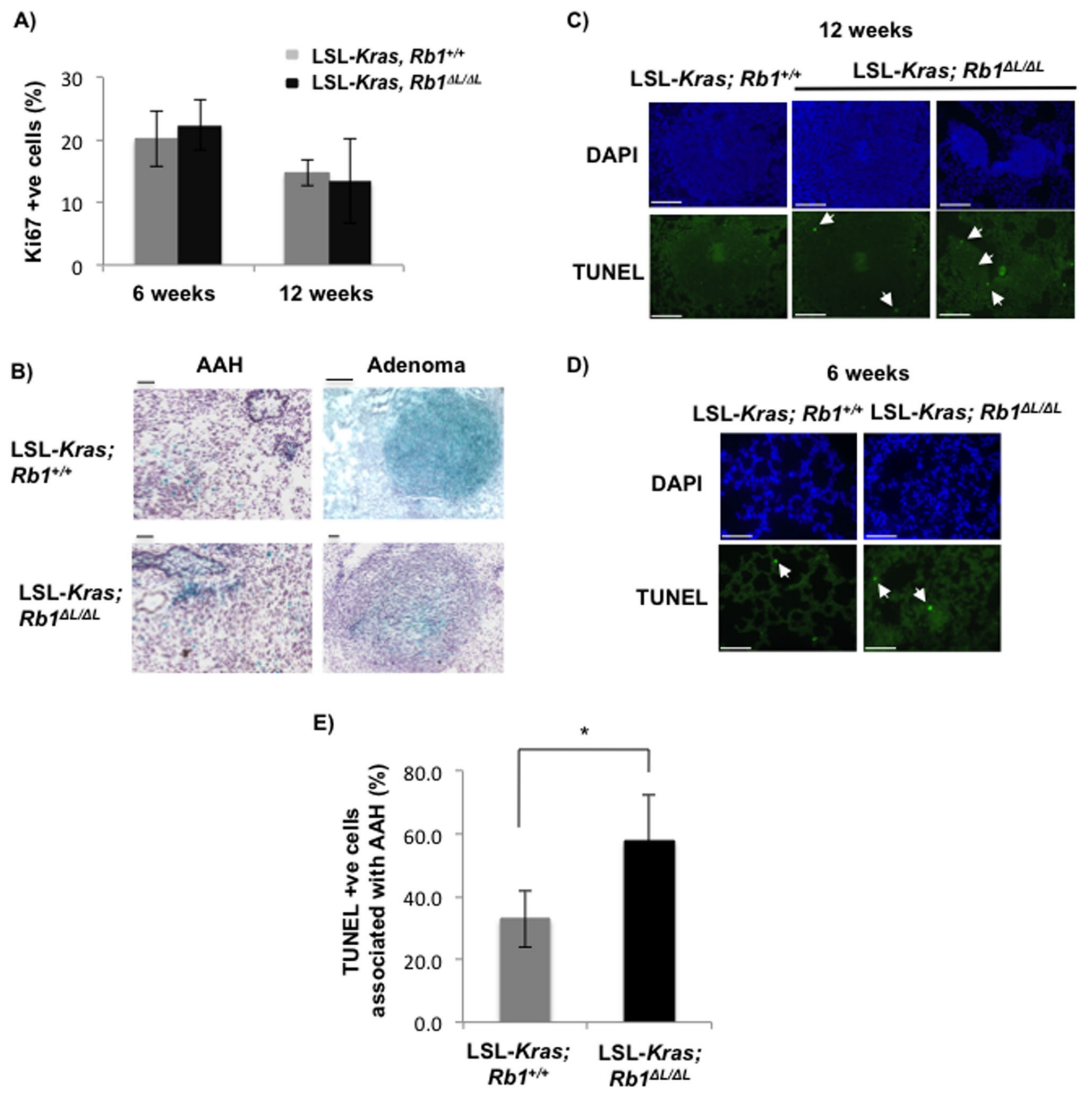
Elevated apoptosis in atypical adenomatous hyperplasia lesions from lungs of *Rb1* ^∆L/∆L^ mice expressing KrasG12D. Six to eight week old LSL-*Kras; Rb1* ^*+/+*^ and LSL-*Kras; Rb1* ^∆L/∆L^ mice were infected intra-nasally with Ad-Cre to activate the oncogenic KrasG12D. Characteristics of lung tumors at six and 12 weeks post Ad-Cre infection were assessed. (**A**) Quantitation of Ki67 positive cells in the adenomas from LSL-*Kras; Rb1* ^*+/+*^ and LSL-*Kras; Rb1* ^∆L/∆L^ lungs at the indicated time points. (**B**) Senescence associated β-galactosidase staining of cryo-sections from LSL-*Kras; Rb1* ^*+/+*^ and LSL-*Kras; Rb1* ^∆L/∆L^ lungs at the indicated time points. Sections were counterstained with haematoxylin. Scale bars are 50 µm. (**C**) Representative TUNEL stained lung sections from LSL-*Kras; Rb1* ^*+/+*^ and LSL-*Kras; Rb1* ^∆L/∆L^ mice 12 weeks following Ad-Cre infection. DAPI was used to counterstain the nuclei. Arrowheads indicate a selection of TUNEL positive nuclei. (**D**) Representative TUNEL stained lung sections six weeks following Ad-Cre infection. DAPI was used to stain the nuclei. (**E**) Quantitation of TUNEL positive cells in B that are associated with atypical adenomatous hyperplasia lesions in LSL-*Kras; Rb1* ^*+/+*^ and LSL-*Kras; Rb1* ^*∆L/∆L*^ lungs. Means were compared by a t-test, statistically significant differences are shown by an asterisk (p=0.001). Scale bars are 50 µm.

In order to investigate the possible cause for the decreased number of adenomas in LSL*-Kras, Rb1* ^*∆L/∆L*^ mice we first tested the proliferation rate of the tumor cells between the two genotypes at six weeks and 12 weeks following the activation of *Kras*. We used Ki67 as a marker for proliferation and SA-β-gal staining as a marker for senescence in the tumors. At both time points tested, the proportion of Ki67 positive cells in the adenomas is similar between the two experimental groups (Figure 5A). This indicates that the tumor cells were proliferating at similar rates in each genotype. Furthermore, we also detected similar senescence staining in the AAH lesions and in adenomas from LSL*-Kras*, *Rb1* ^*+/+*^ and LSL*-Kras, Rb1* ^*∆L/∆L*^ mice (Figure 5B). This further suggested that there is no defect in the proliferation of tumor cells in LSL*-Kras, Rb1* ^*∆L/∆L*^ mice, and activation of oncogenic *Kras* can initiate senescence in these lesions similar to those in LSL*-Kras*, *Rb1* ^*+/+*^ controls.

### Increased apoptosis in atypical adenomatous hyperplasia (AAH) lesions from Rb1^*∆L/∆L*^ lungs expressing oncogenic Kras

Oncogenic ras expression has been shown to induce apoptosis through p53 or pRB-E2F1 pathways [36]. Our *in vitro* results suggested that the clones that escaped senescence have high levels of DNA damage as shown by increased γH2AX staining and activation of the p53-p21 pathway. We wondered if escape from senescence, as a result of the *Rb1* ^*∆L*^ mutation, results in increased cell death by apoptosis *in vivo*. This could potentially explain the reduced number of adenomas that we observe in LSL*-Kras, Rb1* ^*∆L/∆L*^ mice.

We searched for evidence of apoptosis in the benign lung lesions of both *Rb1* genotypes. We performed TUNEL staining on sections from LSL*-Kras*, *Rb1* ^*+/+*^ and LSL*-Kras, Rb1* ^*∆L/∆L*^ mice at different time points following K-rasG12D activation. We noticed very few TUNEL positive cells in the adenomas from the LSL*-Kras*, *Rb1* ^*+/+*^ mice (Figure 5C) at 12 weeks post activation of KrasG12D, suggesting absence of cell death in these lesions. However, some of the adenomas from LSL*-Kras, Rb1* ^*∆L/∆L*^ mice showed TUNEL positive cells at this time point indicating cell death (Figure 5C). We hypothesized that apoptosis might be activated early during tumor development in response to activated ras and defective chromatin regulation by pRB, and that this limits the progression of these lesions. Therefore, we searched for apoptosis in lung sections of mice 6 weeks post activation of *Kras* by TUNEL staining. We counted the TUNEL positive cells from at least 10 random fields from each lung section and quantified how many of these are associated with early hyperplastic lesions (i.e. AAH). We observed a significantly higher number of TUNEL positive cells that are associated with AAH lesions in the LSL*-Kras, Rb1* ^*∆L/∆L*^ mice compared to the controls. This would result in fewer AAH lesions progressing to the adenoma stage and explain why we see lower numbers of adenomas in the LSL*-Kras, Rb1* ^*∆L/∆L*^ mice compared to controls at 12 weeks post activation of KrasG12D.

## Discussion

Our study shows that mutation of the LXCXE binding cleft that disrupts chromatin regulation by pRb is sufficient for escape from oncogene induced senescence and transformation *in vitro*. Interestingly, the pRB^∆L^ mutation does not promote tumorigenesis *in vivo* but instead, reduces tumorigenesis in the KrasG12D lung cancer model by negatively affecting early tumor progression.

We think there are a number of differences between our *in vitro* and *in vivo* experiments that should be considered when interpreting the results of this study. One is the obvious differences in cell types between our experiments. Unlike the lung alveolar pneumocytes, mouse embryonic fibroblasts (MEFs) are not terminally differentiated cells. It is thus possible that MEFs in culture are more readily immortalized whereas additional checkpoints that prevent transformation of the differentiated cells may exist *in vivo*. The increased apoptosis we observe in the primary AAH lesions that are precursors of adenomas and adenocarcinomas suggests this might be the case. It is also interesting to note that MEFs do not undergo apoptosis as robustly, and rather activate senescence in response to persistent DNA damage [27,28,37]. Our data indicates that defective heterochromatinization and deregulation of cell cycle genes during senescence, as a result of the *Rb1* ^*∆L*^ mutation, seems to be sufficient for random cells to escape from this arrest and immortalize *in vitro*. However, the relative contribution of apoptosis and senescence during tumor suppression *in vivo* is not fully understood. Even though both phenomenon are known to be tumor suppressive and are regulated by the same tumor suppressor networks, how and at what stage during tumor progression they coordinate to suppress tumorigenesis remains unknown. We show that senescence is activated in the lungs in response to the activation of oncogenic *Kras* at a very early stage, and benign lesions stain positive for SA β-gal, suggesting that senescence does play a role in suppressing tumor progression in this model. From this perspective it appears that oncogenic *Kras* expression activates both apoptosis and the senescence pathway. Based on our data, cell death by apoptosis and senescence act in series to suppress the progression of benign lesions into malignant adenocarcinomas.

The cause of the increased apoptosis we observed in the LSL*-Kras, Rb1* ^*∆L/∆L*^ mice is not clear. A recent report has suggested a complex interplay between heterochromatin assembly during senescence and suppression of DNA damage response (DDR) signaling [38]. The authors show that disruption of heterochromatin in oncogene expressing cells increases DDR signaling leading to apoptosis. Thus, it is possible that defective heterochromatinization during senescence as a result of the *Rb1* ^*∆L*^ mutation might be exacerbating DDR signaling and induces apoptosis in these lesions early during tumorigenesis. However defective heterochromatinization in mice lacking Suv39h1, the enzyme capable of tri-methylating histone H3K9, show increased susceptibility to lymphoma development in an Eµ-Nras model [10]. This suggests that regulation of heterochromatin by pRb is not equivalent to H3K9 trimethylation and that heterochromatin assembly can have context specific effects on cancer progression.

Our studies using the LSL-*Kras* model and the DMBA chemical carcinogenesis model show that the *Rb1* ^*∆L*^ mutation does not promote tumorigenesis or affect overall survival of the mice. Previous studies done using the *Rb1* ^*∆L/∆L*^ mice also suggested a context specific role for the LXCXE binding cleft during tumorigenesis [39,40]. The *Rb1* ^*∆L*^ mutation co-operates with p53 loss to hasten tumor formation in mice [39]. The tumors in the compound mutant mice are more genomically unstable and the resulting tumors are also more aggressive. Also, in a mammary tumorigenesis model, the *Rb1* ^*∆L*^ mutation exacerbates the tumor phenotype in the Wap-p53 (R172H) transgenic background. However, in the same study it was found that the *Rb1* ^*∆L*^ mutation does not enhance mammary tumors induced by the Neu oncogene. This suggests that tumor suppression by the LXCXE binding cleft of pRb is highly context specific. While the *Rb1* ^*∆L*^ mutation enhances tumorigenesis when combined with p53 loss the same mutation does not cooperate with oncogene activation in the receptor tyrosine kinase (RTK)-ras pathway to promote tumor formation. This might suggest that even though the *Rb1* ^*∆L*^ mutation results in deregulated cell cycle gene expression and defective cycle arrest, *in vivo*, the p53 pathway might act as an additional barrier to suppress tumorigenesis. Increased apoptosis seen in our LSL*-Kras, Rb1* ^*∆L/∆L*^ tumors also seems to support this hypothesis. Tumor progression in these models might require additional disruption of the p53 tumor suppressor pathway. Future experiments using gene targeted mouse models with subtle mutations like ours will help our understanding of the complex relationship between different tumor suppressor and oncogene networks that exist *in vivo*. Identification of incompatibilities, as we have reported here, may offer new therapeutic opportunities in the future.

## Materials and Methods

### Ethics Statement

All animals were housed and handled as approved by the UWO animal use subcommittee (protocol 2011-038) and Canadian Council on Animal Care (CCAC) guidelines. All tumor burdened animals were euthanized when they had reached ethical endpoints.

### Mouse strains

The generation of *Rb1* ^∆L/∆L^ mutant mice has been described previously [26]. LSL-*KrasG12D* mice [18] were obtained from the NCI mouse repository in a B6.129 background and maintained as heterozygotes. Intercrosses with *Rb1* ^*∆L*^ mice (also in B6.129 background), were performed to generate compound mutant mice. Genotyping methods and PCR primers were provided by the suppliers, or are as outlined by Isaac, et al. [26].

### Ad-Cre infection

Ad-Cre was administered by intranasal instillation as described before [18,20]. We infected mice with 5x10^6^ infectious particles of Ad-Cre in 75µl volume per mouse.

### Histology

Lungs were fixed in formalin for 48hrs before embedding in paraffin for staining with Haematoxylin and Eosin. For immunohistochemistry, formalin fixed, paraffin embedded tissues were deparrafinized in xylenes followed by rehydration by serial washes in 100%, 95%, 70% ethanol. Antigen retrieval was done by boiling the sections in a pressure cooker for 15min in 10mM sodium citrate buffer pH 6.0. TUNEL staining was performed using *in situ* cell death detection kit from Roche as per the manufacturers instructions (Cat. No. 11 684 795 910). Tissues for SA β-gal staining were fixed in optimum cutting temperature (OCT) compound and embedded for cryo-sectioning.

### Senescence β-galactosidase staining of tissues

Tissue sections were processed immediately after cryo-sectioning by fixing them in 0.5% glutaraldehyde/PBS for 15 minutes followed by O/N incubation in SA β-gal staining buffer (40mM citric acid/sodium phosphate buffer at pH 6.0 containing 5mM potassium ferricyanide, 5mM potassium ferrocyanide, 2mM MgCl_2_ and 1mg/ml X-gal) at 37^o^C in a humidified chamber. Sections were washed with PBS before sealing with cover slips using Vectamount mounting medium.

### Microscopy

Haematoxylin and Eosin and antibody stained sections are scanned using Aperio Scan Scope CS2 system. Scanned sections were analyzed using Aperio Image Scope viewer software. Lesions were manually counted and graded based on the recommendations of the Nikitin et al. and mouse models of human cancer consortium [41].

### Cell culture

Mouse embryonic fibroblasts (MEFs) were generated from E13.5 embryos using standard procedures and cultured in DMEM with 10% FBS and antibiotics [42]. Retroviral transduction with pBABE-HrasV12 was as reported by Serrano et al. [43] and viruses were packaged in Bosc-23 cells. Cells infected with viruses encoding HrasV12 were selected in 4µg/ml puromycin for at least 3 days before sub-culturing for further experiments. Senescent cells prepared by this method were allowed to senescence for at least 10 days following retroviral infection. Senescence associated β-galactosidase (SA-β-Gal) staining was performed as described before [43]. Isolated foci were continuously sub-cultured in standard medium with 4µg/ml puromycin and passaged every 3 days.

### Soft agar colony forming assay

6 well dishes were coated with a bottom layer of 1.5ml 0.7% low melting agarose in DMEM with 10% FBS and antibiotics. 3x10^4^ cells were resuspended in 1.5ml of 0.35% low melting agarose in DMEM with 10% FBS and antibiotics and added as the top layer to the 6 well plates. All genotypes were tested in triplicate. Cells were allowed to grow in soft agar for 2 weeks before counting the number of colonies formed.

### Antibodies

Anti-γH2AX (05-636) and anti-PML (MAB3738) antibodies are from Millipore. Antiactin (A2066) antibody is from Sigma. Anti-phospho-p53-ser15 (9284) antibody is from Cell Signaling. Anti pan-ras antibody (FL-189) is from Santa Cruz. Anti-p21 (OP-76) antibody was purchased from Calbiochem.

### Immunofluorescence

Cells were fixed in 3% PFA for 10 min at room temperature (RT) and permeablized with 0.5% triton- X-100 for 5 min at room temperature. They were subsequently blocked with 3% BSA/PBS for 15 min at RT followed by incubation with primary antibodies diluted in blocking buffer (1:300 PML, 1:200 γH2AX) overnight at 4^0^C in a humidified chamber. Cells were washed in the blocking buffer 3 times for 5min each. Cells were incubated with Alexa-fluor conjugated secondary antibodies diluted in blocking buffer (1:4000) for 1hr at RT. Cells were washed again 3 times in PBS followed by mounting on slides with mounting medium containing DAPI before analyzing by Confocal microscopy.
